# Factors of surface thermal variation in high-mountain lakes of the Pyrenees

**DOI:** 10.1371/journal.pone.0254702

**Published:** 2021-08-03

**Authors:** Ibor Sabás, Alexandre Miró, Jaume Piera, Jordi Catalan, Lluís Camarero, Teresa Buchaca, Marc Ventura

**Affiliations:** 1 CSIC, Centre for Advanced Studies of Blanes CEAB, Integrative Freshwater Ecology Group, Blanes, Catalonia, Spain; 2 Department of Physical & Technological Oceanography, CSIC, Institute of Marine Sciences, ICM, Barcelona, Spain; 3 CREAF Campus UAB, Edifici C, Cerdanyola Del Valles, Spain; 4 CSIC, Campus UAB, Cerdanyola Del Valles, Spain; University of Maine at Farmington, UNITED STATES

## Abstract

Thermal variables are crucial drivers of biological processes in lakes and ponds. In the current context of climate change, determining which factors better constrain their variation within lake districts become of paramount importance for understanding species distribution and their conservation. In this study, we describe the regional and short-term interannual variability in surface water temperature of high mountain lakes and ponds of the Pyrenees. And, we use mixed regression models to identify key environmental factors and to infer mean and maximum summer temperature, accumulated degree-days, diel temperature ranges and three-days’ oscillation. The study is based on 59 lake-temperature series measured from 2001 to 2014. We found that altitude was the primary explicative factor for accumulated degree-days and mean and maximum temperature. In contrast, lake area showed the most relevant effect on the diel temperature range and temperature oscillations, although diel temperature range was also found to decline with altitude. Furthermore, the morphology of the catchment significantly affected accumulated degree-days and maximum and mean water temperatures. The statistical models developed here were applied to upscale spatially the current thermic conditions across the whole set of lakes and ponds of the Pyrenees.

## Introduction

Temperature is a key environmental variable in lakes and ponds, since it accelerates biochemical reactions, and therefore, increases the rates of many biological and ecological processes including photosynthesis and respiration [1], organic carbon mineralisation [2] organisms’ growth [3], biomass production [4], organisms’ size [5], and ecological processes since it influences thermal niche and species distribution [6], and trophic cascades [7]. More specifically, accumulated degree-days (ADD), which is the temperature integrated in time over a determined threshold, explain organisms’ development [8], while maximum water temperatures limit growth rate [9] and warming tolerance [10]. Temperature range is also essential in explaining life-history traits, as some specialist organisms, occupying a narrow temperature range, present a higher performance [11]. Temperature oscillations, which can operate daily or within different days, are essential because they can increase poikilotherms growth, or can affect them negatively when temperatures are too high [3].

Temperatures have been increasing since the beginning of the industrial revolution, and they are expected to increase between 0.3°C and 4.8°C globally, depending on the scenarios of greenhouse gas emissions, for the 2081 to 2100 period [12]. The air temperature rise translates into a lake water temperature increase [13], although water temperatures trends can vary depending on climatic variables, such as radiation, cloudiness and wind speed, and regional characteristics of the lakes [14], and may differ through the water column of the lakes, showing high increasing trends in shallow waters and low warming trends in deep waters [15,16]. In this sense, water transparency plays an important role in the configuration of the thermal structure of lakes [17–19]. Warming is expected to be greater in both polar regions [20,21] and high mountain areas [22,23]. High mountain areas are globally biodiversity hotspots due to the encapsulation of different climate zones in very short distances [24]. Despite the strong altitudinal gradient, the high spatial heterogeneity of high mountain areas creates a complex mosaic of local conditions, which make the upscaling of the local factors affecting surface water temperatures at regional scales not straightforward, and a missing gap in understanding future scenarios of climate change effects on high mountain species’ distributions. In this regard, the Pyrenees constitute an excellent case study of a high mountain region, containing over 3,000 lakes and ponds. Past air temperatures have been reconstructed in this region at some sites using statistical extrapolations for recent centuries [25,26] or using lake sediment records [27,28]. Also, there have been comparisons between the Pyrenean lakes and other mountain regions in Europe [29]. However, there is not a comprehensive assessment of the variation within the Pyrenean lake district and their associated factors, which could be the basis for a spatial upscaling to the whole region as a reference for ecological studies, particularly of littoral and amphibian organisms. Amphibian populations in Pyrenean lakes need a minimum temperature threshold to develop [3]. Moreover, they are known to be sensitive to water temperature increases [30]. They include species such as the ubiquitous *Rana temporaria* L. 1758 and the endemic species *Calotriton asper* (Dugès, 1852). Surface water warming can also affect macroinvertebrates such as aquatic beetles (Coleoptera, Dytiscidae), which are both sensitive to temperature and predation by introduced salmonids [31]. Fish species as *Salmo trutta* L. 1758, which are invasive to these lakes, have both a minimum and maximum temperature threshold for their development [32]. Both native and invasive species may see their potential habitat altered by increasing temperatures. Therefore, an assessment of thermal conditions of high mountain ecosystems such as Pyrenean lakes are necessary for future studies on ecology and conservation in these ecosystems.

Many studies have dealt with thermic variables monitoring and modelling of lake surface water temperature (LSWT) at different temporal scales: daily [13,33,34], monthly [35] or seasonal [36,37], maximum annual temperature [38], daily minima and maxima [39] and diel temperature range (DTR) [40,41]. Also, accumulated degree-days, and temperature oscillations have been used for physiological studies of freshwater organisms [3,8], and accumulated degree-days have also been modelled [8,42]. However, no studies have attempted to develop models of a handful of thermic variables using the same methodology.

The physical processes that drive the heat balance in lakes are essentially known [43]. However, the available physical models require the input of driving variables which are hardly available for a large number of lakes. Therefore, the upscaling of thermal conditions to large lake districts is difficult. Statistical modelling is an alternative that requires a sufficiently large calibration set and the identification of the main landscape factors that primarily constrain the actual physical drivers. We used surface water temperature 9-year series from 59 lakes and developed mixed regression models. We included explicative factors that constrain the atmosphere-water thermal interaction due to the location (latitude, longitude and altitude), the system inertia (lake area), the advective heat flow (water renewal and hydrological complexity of the catchment), and the incoming radiation (topography). These constraints do not consider interannual variation but mean conditions; therefore, we also used spring and summer air temperatures from a weather station in the Central Pyrenees to account for this temporal variation. We also modelled some thermal niche features that are commonly affecting a variety of organisms, namely ice-free period mean and maximum temperature, accumulated degree-days, mean diel temperature range, and temperature oscillation; the latter defined as the difference in maximum temperature between a time-lapse of three days. Warming can increase habitat availability (e.g., accumulated degree-days in cold lakes) and limit species survival (e.g., maximum temperature in warm lakes).

## Methods

### Temperature and environmental parameters

**Fig 1:**
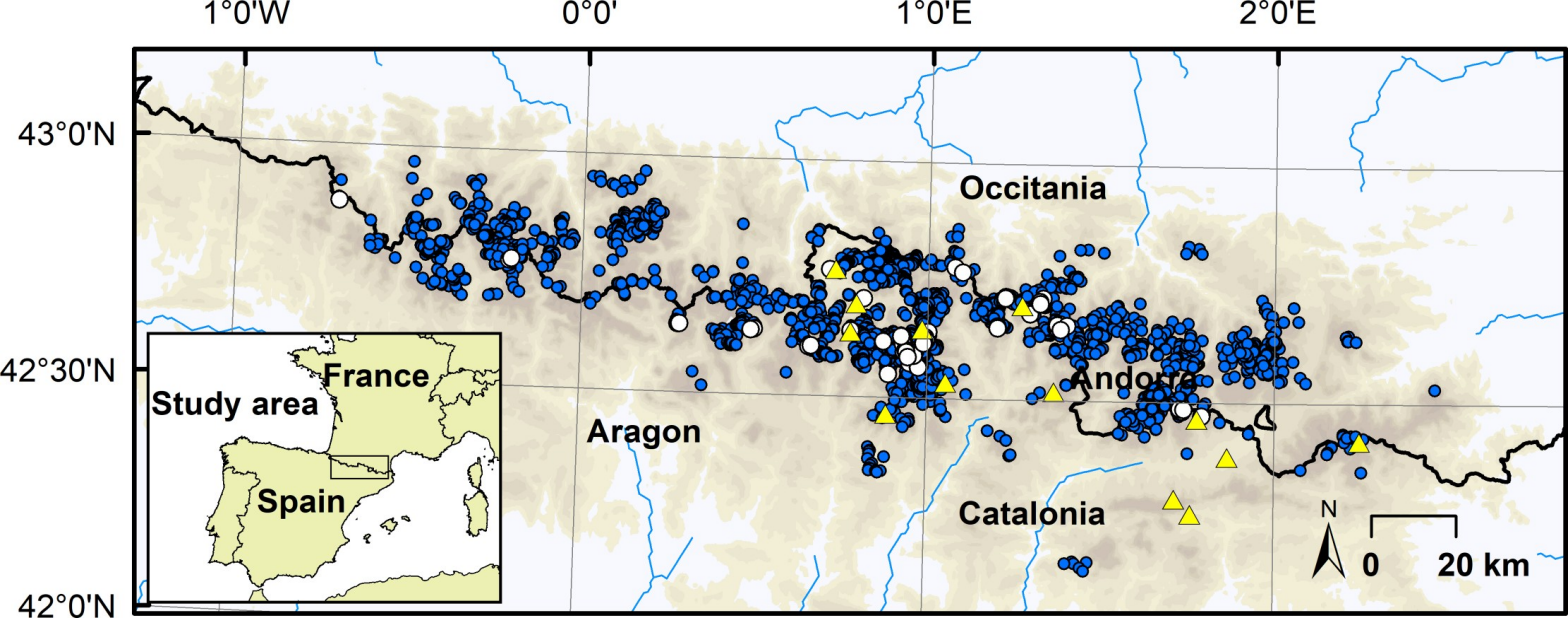
Map of the studied Pyrenean lakes.

A set of 59 lakes and ponds were selected to deploy minilog-thermistors with attached dataloggers (Vemco Minilog-T) with a precision of ±0.1°C spread along the Central—Eastern Pyrenees (42 and 43° N and between 1° W and 3° E; Fig 1), to consider a wide range of water temperature variability. The lakes and ponds were selected following key environmental variables, which can affect high mountain lake water temperatures, such as altitude, lake and catchment size, residence time, and radiation (See S1 Table and S1 Fig for a description of their ranges and comparison with the Pyrenean water bodies). Temperature thermistors were deployed in the lakes of the National Park of Aigüestortes i Estany de Sant Maurici, the Natural Park of Alt Pirineu and Natural Park of Posets-Maladeta with permissions from the park authorities. Other lakes belonging to public domain did not require other specific permissions (those belonging to protected sites are detailed in S2 Table). No endangered or protected species were involved in this study. Thermistors recorded temperature continually, all year long, and were replaced before batteries depleted. They provided us with complete summer water temperatures for 2001, 2002, 2004 just in Lake Redon, and from 2009 to 2014 for the whole set of water bodies, constituting 9 years of recorded summer water temperatures. Thermistors were deployed from the lakeshore at 1.5 m depth and separated from the lake bottom using a fishing rod. Temperature measurements were taken at 90-minute intervals. The thermic variables were calculated for the ice-free periods, defined as the periods over 4.0°C. Mean temperature (Tmean) was calculated as daily mean temperatures averaged over that period. Maximum temperatures (Tmax) was the maximum recorded each year. Diel temperature range (DTR) was calculated as the average of diel temperature ranges over the ice-free period, and temperature oscillation (Tosc) was calculated as the difference in maximum temperatures in a three-day lapse, averaged over the same period. The whole set of thermal variables for each lake and year are detailed in S2 Table.

The lakes and ponds with measured water temperature are represented in white. All the mapped lakes and ponds of the region are in blue and the automatic weather stations are in yellow triangles.

ADD were calculated over two different temperature thresholds (T_T_), 4.0°C, and 7.6°C, the first (ADD4) representative of the ice-free period, and the latter (ADD7.6) as an animal development threshold, as it was the minimum temperature necessary for the development of *Rana temporaria* larvae [3]. For ADD, but not for the other thermic variables, the variables were transformed to ADD at 0.1m depth as it is more representative of the habitat of these organisms, the transformation was performed using a linear regression between ADD at 1.5m and ADD at 0.1 m in 5 lakes. See S1 Section for a detailed description of the ADD calculation procedures.

Mean spring (March–May) and summer (June–August) air temperatures were calculated for years 2000 to 2015 from available daily data of the Catalan Meteorological Service in 14 altitude automatic weather stations located in the Catalan Pyrenees (Fig 1; S3 Table). Air temperatures used in the models were those of Lake Redon meteorological station, which is located in a central location in the Pyrenees and has a long temperature series, given that all temperature series were highly correlated (S2 Fig) and were representative of each year seasonal air temperatures in the region. Other meteorological variables, which are known to affect water temperatures, such as wind, precipitation or cloud cover, were not considered in this study, as they are less coherent spatially in a mountain region as the Pyrenees.

Lake surface area (Larea), direct catchment area (Dcatchment), which is the area where water flows directly to the water body (i.e. not passing through another lake), and total catchment (Tcatchment) were digitalised and calculated following existing topographical cartography and aerial photography from the Catalan Cartographic and Geologic Institute, the French National Geographic Institute and the Spanish National Geographic Institute, as it was done by Casals-Carrasco et al. [44]. From these variables, we calculated two ratios describing the geomorphology of the catchments; Dcatchment/Tcatchment, which is inversely related to water retention in the upper lakes of the catchment, and Tcatchment/Larea, which is a surrogate of water renewal time, given comparable precipitation among sites and time, since runoff is related to catchment size [45] and lake depth is related to lake area [46]. So lake volume is proportional to lake area. Since water renewal time is defined as the ratio between runoff and lake volume it can be represented by Tcatchment/Larea. Geographic coordinates were expressed as X and Y coordinates in ETRS89 UTM coordinate system in the zone 31N. Theoretical radiation data for each lake and its catchment were calculated from a digital elevation model (15 m of pixel resolution) of the Pyrenees. We calculated the monthly radiation using the Area Solar Radiation algorithm [47]. This analysis tool calculates insolation across a landscape for specific locations based on methods from the hemispherical viewshed [47]. Global, direct and diffuse radiation and the sun hours were calculated for both the lake surface and the whole catchment. Radiation was calculated considering a solar constant of 1,367 W m^-2^, considering solar track, atmosphere attenuation and topography (defined with the digital elevation model). Radiation variables were highly correlated among them (S3 Fig). Therefore, we chose the sun hours in the total catchment as a representative of them all, because in a preliminary analysis it was the radiation variable with a better statistical performance.

## Statistical modelling

Mixed models were performed in R language version 3.6.1 [48]. They were constructed relating thermic variables (ADD7.6, ADD4, Tmean, Tmax, DTR, and Tosc) to altitude, lake area, geomorphology, X and Y coordinates, radiation (as the sun hours in the total catchment) and air temperatures of spring (Tspring) and summer (Tsummer), since they are the previous and current season of the ice-free season. The geomorphology variables included the ratio between direct and total catchment (Dcatchment/Tcatchment) and the ratio between total catchment and lake surface area (Tcatchment/Larea) as a surrogate of lake water renewal time. These two ratios and Larea were normalised by logarithmic transformation. We checked that correlation between pairs of explicative variables was not too high (r <0.71) to avoid collinearity effects (S4 Fig). All variables were standardised to z-scores subtracting the mean and dividing by the standard deviation of these variables in the 59 sampled lakes during the 9 studied years. Random variables considered in the models were year and waterbody type (classified as pond <0.5 ha and lake >0.5 ha).

The model selection used followed the ten-step protocol described by Zuur et al. [49]. It consisted of a first selection of the variables in the fixed part, first adjusting ordinary multiple linear regression models, then, selecting variables by stepwise selection, choosing the model with the minimum Akaike Information Criterion (AIC). We considered two different model selection methods for each variable, using forward or backward selection. We also considered interactions between the most relevant variables (Altitude, Larea, Tspring and Tsummer). Later, we adjusted mixed models with random structures, by restricted maximum likelihood (REML), using the “lme” package [50] and we compared them with the ordinary multiple regression model. The null random structure was tested with three different structures: the year, the water body type and the year nested in the water body type. The structure with the lowest AIC was selected. The following step was to test the optimal fixed structure using the likelihood ratio test with the mixed model estimated with maximum likelihood (ML) to determine the significance of the variables and drop the non-significant ones. Finally, the model was refitted with REML and tested for homogeneity of variance and independence. From the four models calculated for each variable, the one with the lowest AIC was kept (all models tested are in S4 Table). Summary statistics of R^2^ marginal (R^2^_m_) and R^2^ conditional (R^2^_c_) were calculated with the package MuMIn [51], where R^2^_m_ concerned the fixed part of the model, while R^2^c referred to both the fixed and the random part. When the resulting model had no random part ordinary R^2^ (R^2^_o_), and adjusted R^2^ (R^2^_adj_) were calculated, the latter penalised by the number of coefficients. RMSE calculation was performed using k-fold cross-validation of the models (k = 5).

As the modelled water temperature variables are of great interest for the biology and ecology of lake organisms along the entire region, we used the models presented here to make present projections along the Pyrenees in a wide dataset of 2,267 lakes and ponds. To do so, we multiplied the coefficients of the mixed models with the spatial data of these water bodies and the seasonal air temperature in Lake Redon AWS for the period between 2006 and 2015.

## Results

### Thermic characteristics of Pyrenean lakes and ponds

**Fig 2:**
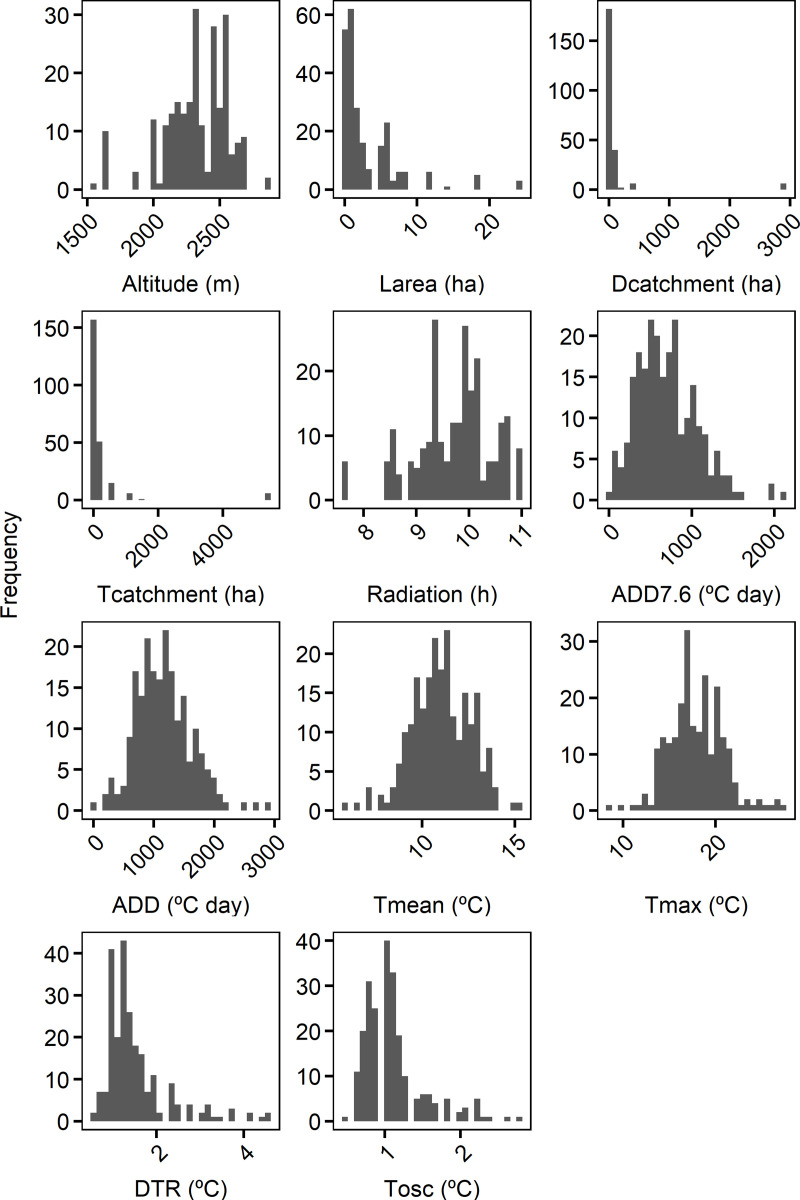
Frequency distribution of the morphological, radiation and thermic variables calculated in the 59 studied water bodies. See Table 1 for the description of the variables. Here the variables are not transformed.

Thermic variables showed a wide range in the monitored Pyrenean water bodies during the ice-free period (Fig 2, S1 Table). Tmean ranged between 5.9 and 15.3°C, being 11.0°C the average temperature for all water bodies. Tmax reached a value of 27.3°C, while the lowest Tmax registered was only 8.4°C, having a mean Tmax of 17.9°C. DTR had a mean of 1.5°C and ranged between 0.6 and 4.6°C. Tosc (maximum: 2.8°C) was smaller than the maximum DTR, but was similar for the mean and minimum (1.1 and 0.5°C respectively). Some water bodies had low ADD, having a minimum of 18.1°C day ADD7.6 and 41.6°C day ADD4, while mean ADD (697.2 ADD7.6 and 1,161.8°C day for ADD4) and maximum ADD (2,126.5°C day for ADD7.6 and 2,931.5°C day for ADD4) were much greater.

### Models of thermic variables

**Fig 3:**
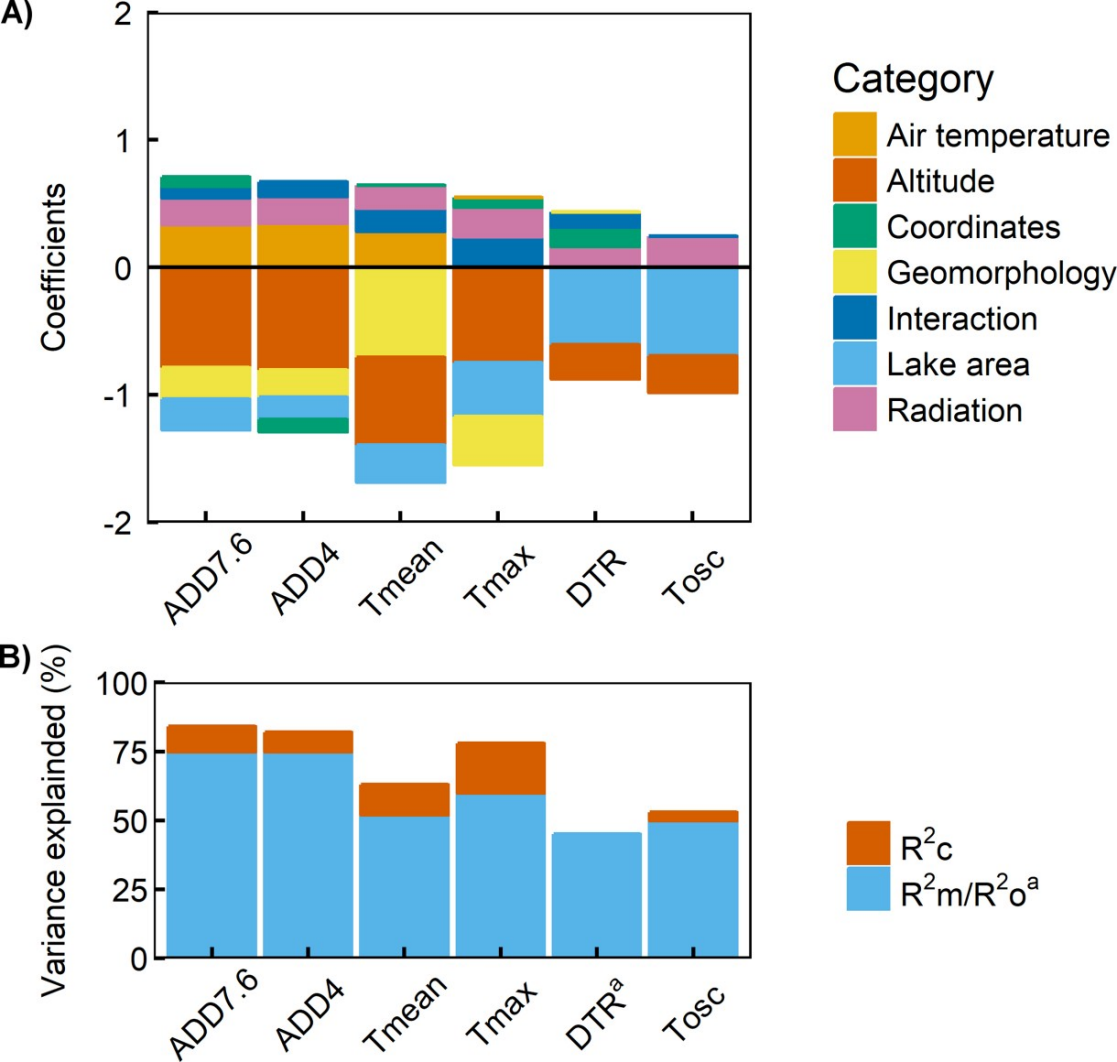
Mixed regression models. A) Coefficients of the fixed variables of the models classified in six categories plus the interactions. Where Air temperature includes Tspring and Tsummer, Coordinates are X and Y and Geomorphology includes Dcatchment/Tcatchment and Tcatchment/Larea (See Table 1 for variables description). B) Conditional (R^2^_c_) and marginal (R^2^_m_)/ordinary (R^2^_o_)^a^ R^2^ of the models. Conditional R^2^ account for the random variability of the model, whereas marginal R^2^ account for the fixed part, ordinary R^2^ is depicted in the case of multiple regression.

Mixed models of thermic variables showed good fits (Fig 3B, Table 1; R^2^_c_, 0.53–0.84). Only the model for DTR fitted better without the random part in the model, and therefore, it was adjusted as a multiple regression model and resulted in a lower fit (R^2^_o_ 0.45). The rest of the models included the year as a random variable except Tmean, which only included the water body type (lake or pond). The water body type was also included in the model for ADD7.6 and ADD4 together with the year.

**Table 1:** Summary of the coefficients of the mixed models.

	ADD7.6	ADD4	Tmean	Tmax	DTR	Tosc
Intercept	-0.06	-0.06	-0.06	-0.10	0.04	0.00
Radiation	0.21	0.21	0.18	0.23	0.16	0.24
Dcatchment/Tcatchment			-0.23	-0.09		
Tcatchment/Larea	-0.25	-0.21	-0.48	-0.28		
X	0.07			0.07	0.15	
Y		-0.09				
Larea	-0.24	-0.18	-0.29	-0.42	-0.61	-0.69
Altitude	-0.79	-0.81	-0.69	-0.75	-0.27	-0.29
Tspring	0.34	0.35	0.16			
Tsummer			0.12			
Larea:Altitude	0.14	0.12	0.18	0.16	0.12	
Altitude:Tspring	-0.05			0.08		
sd fixed	0.87	0.86	0.75	0.79		0.71
sd water body	0.00	0.02	0.34			
sd year	0.31	0.28		0.43		0.17
sd residual	0.40	0.42	0.63	0.48	0.75	0.68
R^2^ marginal/ordinary^a^	0.75	0.75	0.52	0.60	0.45^a^	0.50
R^2^ conditional/adjusted^a^	0.84	0.82	0.63	0.78	0.44^a^	0.53
AIC	322.45	299.83	493.11	399.08	536.14	507.80
BIC	363.39	336.06	530.39	436.61	560.29	528.32
RMSE	181.31	237.14	1.21	1.89	0.55	0.68

The coefficients of the models are calculated from z-scores obtained from the original values. The coefficients of the fixed part are Radiation: Sun hours in the total catchment (hours per day); Dcatchment: Direct catchment area (ha); Tcatchment: Total catchment area (ha); Dcatchment/Tcatchment: Logarithm of the direct catchment area/total catchment area ratio; Tcatchment/Larea: Logarithm of the total catchment area/lake area ratio; X: X ETRS89 UTM 31N coordinates (m); Y: Y ETRS89 UTM 31N coordinates (m); Larea: Logarithm of water body area (ha); Altitude (ha); Tspring: Spring air temperature (°C); Tsummer: Summer air temperature (°C); their interactions (Larea:Altitude and Altitude:Tspring) and the standard deviations of the models: sd fixed: Standard deviation of the fixed term; sd water body: Standard deviation due to the water body type; sd year: Standard deviation due to the year; sd residuals: Standard deviations of the residuals, R^2^ conditional and R^2^ marginal for mixed models (having both fixed and random part) and R^2^ ordinary and R^2^ adjusted for multiple regression models^a^ (only with the fixed part) are represented for seven thermal variables of the lake: ADD7.6: Accumulated degree-days over 7.6°C (°C day); ADD4: Accumulated degree-days over 4.0°C (°C day); Tmean: Mean epilimnetic temperature of the ice free period (°C); Tmax: Maximum epilimnetic temperature (°C); DTR: Diel temperature range (°C); Tosc: Oscillation in maximum temperature in a three-day lapse (°C).

The models for ADD7.6 and ADD4 showed the best performance (R^2^_c_ 0.84–0.82). They were mostly explained by the fixed part of the model; R^2^_m_ was the highest of all the models (0.75) (Fig 3B, Table 1). The Tmean model had the lowest adjustment for the fixed part (R^2^_m_ 0.52), while Tmax had the most important effect of year and water body (Fig 3B). Tosc had the lowest performance among the mixed models (R^2^_c_ 0.53).

**Fig 4:**
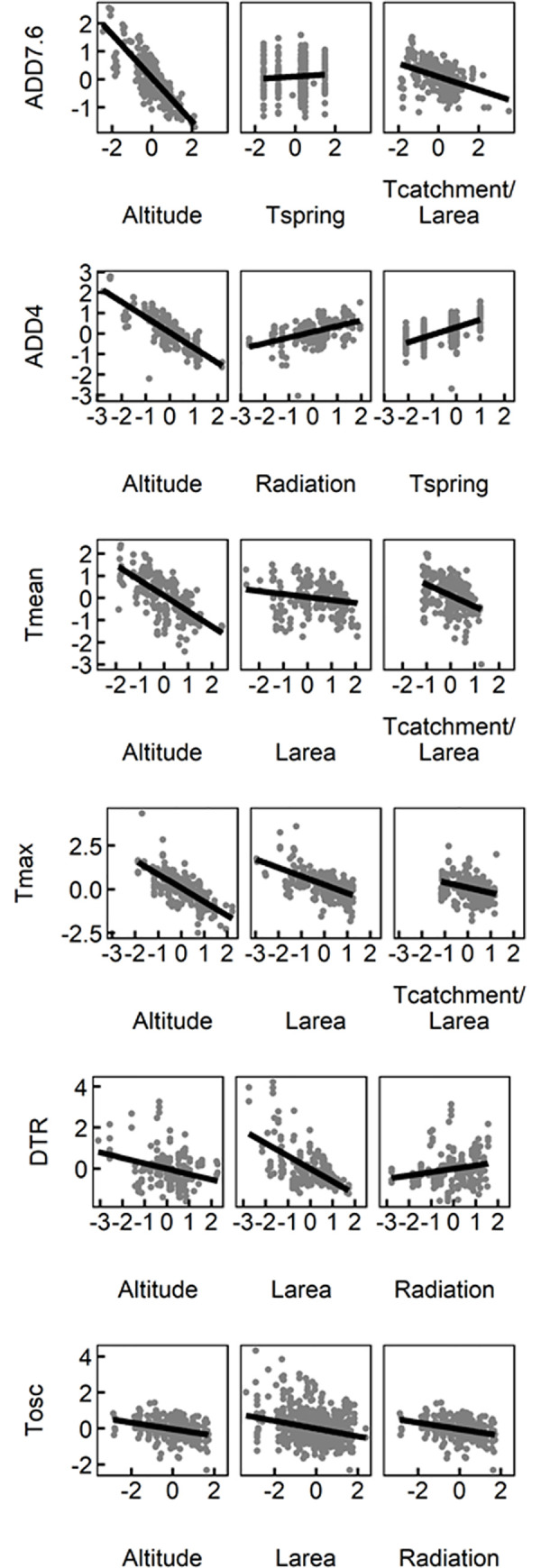
Partial effects of the explicative factors on modelled thermic variables. Only the three variables with higher influence in the models are drawn (See Table 1 for the complete model description).

The primary variable explaining the variation in thermic variables was altitude, whose mixed models coefficients were between -0.27 and -0.81 depending on the response variable (Fig 3A; Table 1): Altitude showed an inverse relationship with all thermic variables; it affected more ADD7.6, ADD4 and Tmax and it had a lower effect on DTR and Tosc (Fig 4). In contrast, lake area was more important than altitude in explaining DTR and Tosc (-0.61 and -0.69 respectively) (Fig 3A; Table 1). It also had an inverse relationship with all variables (Fig 4). Larea was also important in explaining the maximum temperature (-0.42) (Fig 3A; Table 1). Geomorphology had its most important role in mean temperature (-0.71), but it also affected ADD7.6, ADD4 and Tmax (-0.21 - -0.38) (Fig 3A; Table 1). Greater Tcatchment/Larea and Dcatchment/Tcatchment produced a decline in water temperature, and therefore, an inverse relationship with ADD7.6, ADD4, Tmean and Tmax but not on DTR or Tosc (Fig 4). Tcatchment/Larea can be considered as a surrogate for water renewal time. This variable explained most of the effects of geomorphological variables. Radiation had a similar positive effect on all thermic variables (0.16–0.26). Geographic coordinates had a limited effect (-0.09–0.15), with an increase of ADD7.6, Tmax and DTR to the west and a decrease of ADD4 to the north.

Interannual variability in air temperatures, represented by the seasonal air temperatures in Lake Redon, had a substantial positive influence on ADD7.6, ADD4 and Tmean positive interaction with altitude on Tmax, and no effect on DTR and Tosc. Of the two seasons considered in the models, spring and summer, spring showed the strongest effects of air temperature on the thermic variables (Table 1). Interannual variability in air temperature accounted for a lower effect than the sum of the other variables which depended on the lake characteristics, such as altitude, lake area, morphology or radiation (Fig 3B). Nevertheless, it has to be taken into account that the variability attributed to the year inside the random fraction of the models represents interannual variation due to other undetermined meteorological causes out of mean air temperature, which would include wind, precipitation, and cloudiness.

### Projections of thermic variables

**Fig 5:**
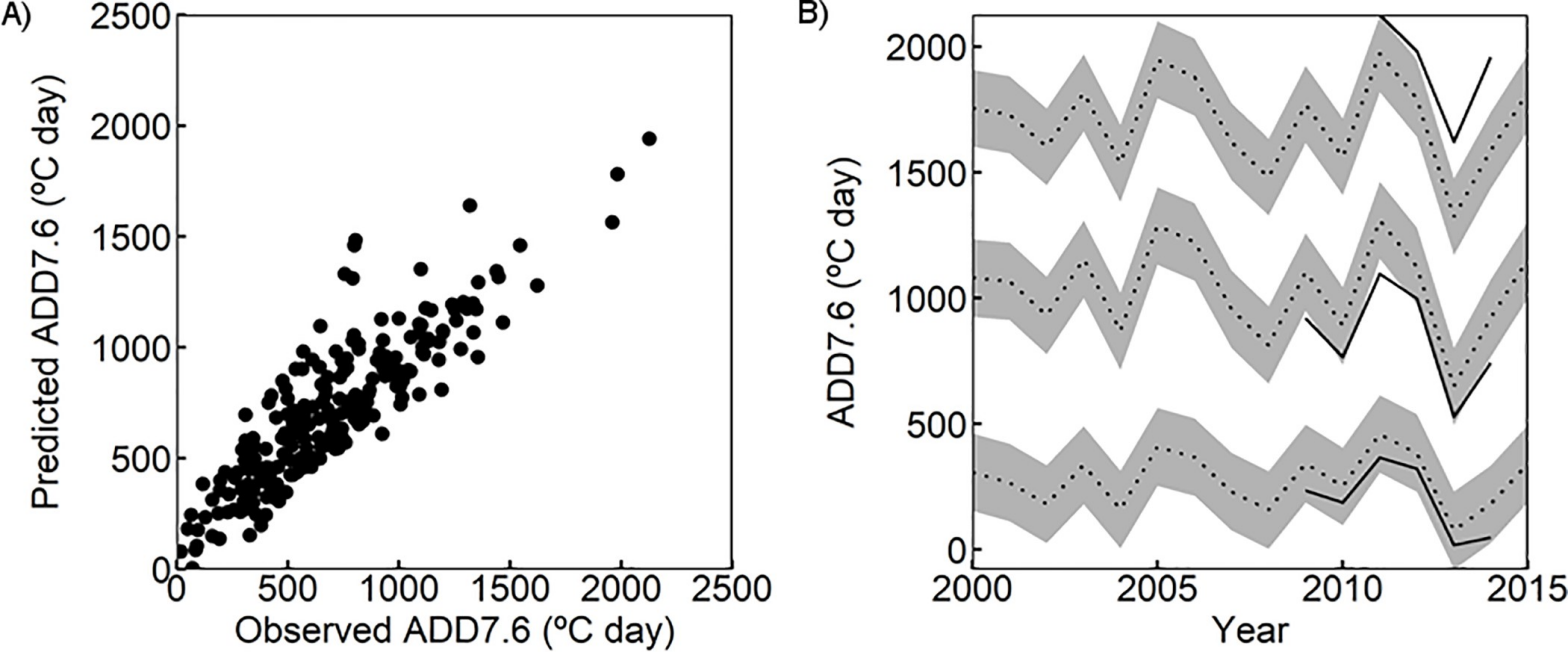
Predictions of ADD7.6 models. A) Predicted against observed ADD7.6. Predicted ADD7.6 are calculated from averaged predictions of the cross-validation (k = 5). B) Observed and predicted ADD7.6 (°C day) for lakes Estanyet de Sotllo, Llebreta and Estanho de Vilac (from colder to warmer). Observed values are depicted with continuous lines, while predicted values are drawn with dotted lines. Standard deviations to the predicted values are represented by grey ribbons.

Projections in the lakes and ponds for the measured years using k-fold cross-validation provided a value of RMSE for the models (Table 1). Predicted values were in good agreement with the observed ones (Fig 5A). Predictions for ADD had lower errors than for Tmax and Tmean while DTR and Tosc showed higher prediction errors. Predicted time series followed the same temporal pattern as observed thermic variables and generally observed values were within the error of the predicted thermic variables or close to it. Biases of the predictions were related to extreme events and to the fact that DTR and Tosc were not explained by interannual variability in air temperatures (Fig 5B).

**Fig 6:**
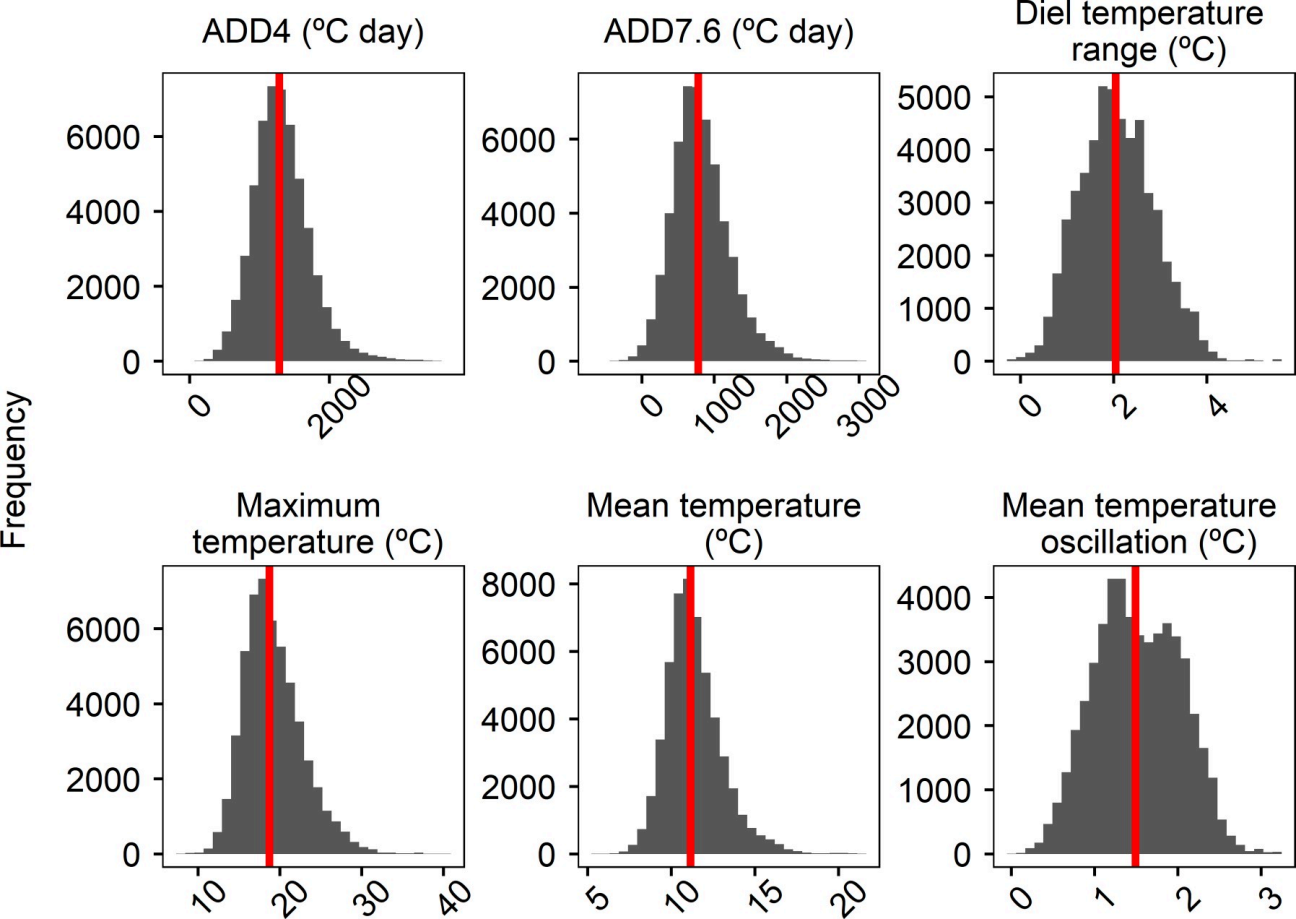
Projections of thermal variables for the Pyrenean lakes (n = 2,267) during the period between 2000 and 2019.

These models allowed us to project the thermic variables for the whole set of lakes and ponds along the Pyrenees (n = 2,267) during the time of this study, using measured seasonal air temperatures in Lake Redon AWS from 2000 to 2019 and the spatial information about these water bodies. Mean seasonal air temperature in Lake Redon was 0.77°C in spring and 10.55°C in summer. Maximum mean temperature in spring was 2.62°C in 2005 and, in summer, 13.47°C in 2003. Projections for lakes and ponds for these two decades are included in S5 and Fig 6. In average, for all these water bodies Tmean was 11.36°C, ADD7.6 was 820°C days, while ADD4 was 1,315°C days, Tmax was 19.21°C, DTR was 2.08°C and Tosc was 1.52°C. Maximum ADD7.6 projected in Pyrenean lakes was 3,09°C days and ADD4 was 3,670°C days. Maximum Tmean in those lakes was 21.55°C, while maximum Tmax was 40.78°C, which was far beyond measured maximum temperatures. These extreme values were found in six small (8–63 m^2^) and low altitude ponds (1479–1694 m). Maximum projected DTR was 5.58°C and Tosc was 3.01°C. Minimum Tmean was 5.64°C, and minimum Tmax was 7.57°C. There were 107 lakes with negative ADD7.6, which were at altitudes between 2362 and 2978 m.a.s.l. and one outlier with negative ADD4, which was the highest lake. Conceptually, negative ADD are not possible, but they are a result of extrapolations in extremely cold lakes, which would have ADD close to zero. Also three lakes showed negative DTR which were the three big lakes, with areas between 41 and 88 ha. Minimum Tosc was 0.017°C.

Histograms of the projections are represented for ADD7.6, ADD4, Tmean, Tmax, DTR and Tosc. Median values are represented with red lines. See Table 1 for the description of the variables. Here the variables are not transformed.

## Discussion

Among all the predictor variables, altitude was the factor that explained more variability in lake surface water temperature in the Pyrenean mountain range, since lake surface water temperature decreases linearly with altitude as the air temperature adiabatic lapse rate, but at a higher rate, as found in the Alps [13]. This was the case for accumulated degree days, mean and maximum water temperatures. This relationship has been previously described in lake districts situated in mountain ranges at daily and monthly scales [13,34]. In the case of maximum temperature, a weak relation with altitude was found in Canadian lakes [38], probably because they considered a reduced altitude range compared to ours (1053–2978 m; S1 Table). In contrast, we found that altitude was the main explicative variable for maximum temperatures in high mountain lakes. Previous studies have successfully modelled accumulated degree days in lowland areas (Midwest) of North America [42]. In this study, we show the relevance of altitude on accumulated degree-days in mountain areas.

In the case of diel temperature range and temperature oscillation, lake area was the most explicative variable. This was also found by Woolway et al. [41], and it is a result of lake area being proportional to lake depth and mixing depth [46,52], which increase thermic inertia, and therefore, reduce diel temperature range [53]. Besides, we have found that, in lakes located in high mountain ranges, altitude was also a significant variable explaining differences in diel temperature range together with other less importance variables, as longitude and insolation (Fig 3). Lake area was also relevant to model accumulated degree-days, mean and maximum temperatures. Similarly to our results, but at daily scales, lake area smoothed water temperature comparing to that of air [33], as the lake morphometry is related to the heat exchange between water and air and the heat storage.

In addition to lake area, morphological variables related to lake catchment features were found to be important for modelling water temperature. The total catchment to lake area ratio can be used as a proxy of the inverse of water renewal time, and consequently, higher ratios corresponded to lower accumulated degree-days, mean and maximum temperature of the water bodies, as faster flushing and increased water coming from snowmelt would prevent a warming up of the epilimnion of the lakes and ponds. The ratio between direct and total catchment may be inversely related to water retention in upstream lakes and ponds and increased heating in these water bodies, and thus higher ratios corresponded to lower water mean and maximum temperatures. Taking into account these variables may help us to understand the potential effects of precipitation on lakes’ water temperatures, as catchment morphology variables have not been considered in the development of empirical models of water temperature. In the case of precipitation, it is commonly used in mechanistic models of water temperature [42], but it is rarely used in empirical ones, and effects of precipitation were not even found [38]. In the Mediterranean region, precipitation is expected to decrease (IPCC, 2013), which could lead to lower water renewal times resulting in even higher warming in lakes. Testing the effects of precipitation in conjunction with the catchment morphometries could help us understand the thermic characteristics of lakes. This would require a good understanding of the precipitation spatial and temporal distribution.

Solar radiation, as sun hours reaching the water bodies’ catchment, had a notable positive effect on lake temperature. In our study, we focused on the local differences in solar radiation instead of seeking temporal variations. Spatial modelling allowed us to find the effects of latitude, altitude and the shading by topography on incoming solar radiation into the lakes and catchments, and thus on thermic variables of the lakes. It is already known that the effect of shading by topography was of great importance in high mountain ranges, where shaded lakes were cooler than expected by their altitude [13]. Different approximations have been considered when introducing solar radiation into water temperature models. For instance, theoretical clear-sky solar radiation has been used to model daily water temperatures in Greenland [33], where altitude and latitude were used, but not the shading by topography. Another option is the use of radiation downscaled from global climate models (GCMs) [42], in such an approximation, spatial differences can be assessed although topographical shading may not be considered. Using solar radiation in empirical lake surface water temperature models showed contrasting results. Whereas solar radiation was not a good predictor for maximum temperature in Sharma et al. [38], spatial and temporal changes in solar radiation were related to lake warming trends worldwide using satellite data [14]. The spatial variability in incoming solar radiation can vary from one study to another, as latitude, altitude and shading depend on the scale and the region studied. In the case of the Pyrenees, latitude might not be very relevant as the mountain range is oriented W-E, but altitude and topographical shading have an important effect on spatial variability of incoming solar radiation. Whereas radiation derived from GCMs, or from satellite data are a good option to account for temporal and local variability in solar radiation, our work shows the important effects of topography on mountain regions, when modelling water temperatures, as it has a great spatial precision, and it is commonly neglected. Integrating methods to account for more precise temporal and spatial calculations of radiation arises as an excellent option to further improve models like the ones presented here.

Interannual variability in air temperatures was represented by the seasonal temperature data series of Lake Redon, which represented air variation at a regional scale. This temporal change in air temperatures explained accumulated degree-days and mean and maximum temperatures in higher altitudes, whereas it did not explain diel temperature range or temperature oscillation. This may be related to a higher unpredictability of the last three variables (Fig 3B; Table 1), and due to a probable increase in minimum water temperatures during summer, for diel temperature range and temperature oscillation. Spring air temperatures explained more variability of the thermic variables than summer temperatures. The latter was only significant for mean water temperature. This may be due to spring temperature effect on thawing and ice-off and the onset of lake surface water temperature warming, as in the Tatra mountains, where the lake surface water temperature began to show an altitudinal gradient in late spring [34]. Mean annual temperature, in addition to summer temperature, was significant to explain water maximum temperatures in Canada [38], indicating that air temperature is influencing lake surface water temperature also beyond summer, especially during spring. Other variables which can contribute to the interannual variability of the thermic variables are wind, precipitation and cloudiness [42], which were not explicitly included in these models, but are represented by the random variability due to the year. This variability is especially high in the case of maximum temperatures. Future research can be conducted to disentangle the effects of these meteorological variables, as their degree of coherence is lower than for air temperatures, so their spatial variation has to be well known before considering them in predictive models.

Lakes and ponds at low altitudes would show a greater increase in accumulated degree-days over the 7.6°C threshold in response to spring temperature increase, as we found a negative interaction between altitude and spring air temperature on accumulated degree days (Table 1), which meant contrasting effects of interannual air temperature on higher and lower lakes and ponds. This was likely a result of a greater advance in ice-off date at lower than at higher altitude, causing high altitude lakes to be disconnected during more time from spring air temperature, as ice-cover has an insulating effect [34]. In contrast, maximum water temperatures, which take place at the middle of summer, are expected to increase more at lakes from higher altitude, since they depended on the positive interaction between interannual air temperature and altitude (Table 1). This may be the consequence of a greater increase in air temperatures in altitude, as described in a review by Pepin et al. [54]. Also, accumulated degree-days over 7.6°C threshold in ponds (surface areas < 0.5 ha) had a steeper decreasing slope with altitude than in lakes (> 0.5 ha), as lake area interacted positively with altitude on accumulated degree-days, an interaction which was also found for mean and maximum temperatures and diel temperature range (Table 1). Low altitude ponds may accumulate more heat because of earlier ice-off and a quicker response to air temperature rise in comparison with low lakes. High altitude ponds, in turn, may be more affected by cooling in autumn, whereas lakes may remain warmer at high altitudes because of higher thermic inertia. Besides, differing warming trends between small and big lakes have been described, as small lakes benefit of higher wind sheltering, smaller fetch, and thus they may show stronger stratification and a warming trend in the epilimnion and smaller or opposed trend in the hypolimnion [15]. Furthermore, the transparency of the lakes has a primary role in conforming the thermal structure of the lake, as the radiation is absorbed in the surface of the lake enhancing the stratification strength, and increasing radiative loss to the atmosphere. This process is especially relevant in small lakes, where wind stirring has less importance [18]. Differences between thermal characteristics of lakes and ponds are relevant as these habitats can have different species composition, as has been found between lakes and pond from the Pyrenees [55]. Lake thermal structure is also relevant as it will affect differently the species depending at which depth they inhabit. Therefore, a deeper knowledge on different warming trends is fundamental to assess potential effects on the organisms of these ecosystems. In the Pyrenees, further research on the thermal sturcture, the hypolimnion trends, the differences in warming between ponds and lakes and the processes involved should be done to completely assess the effects of temperature changes on the whole ecosystem.

We have shown that, for a nine-year period, the effects of variables which depend on the spatial distribution of the lakes (altitude, lake area, catchment morphology and radiation) were more important than the effects of interannual air temperature differences for all thermic variables. These results may vary in a wider time window, as the measured period was of slight temperature increase in comparison to the last half-century (S2 Fig), when temperature had a steeper increase [56], and may continue to do so with the current scenario of climate change, thus rising upper temperatures [12,57]. However, mean summer air temperatures from this study ranged between 8.2 and 11.3°C, while it was found to be between 6.1 and 11.6°C from 1781 to 1997 in the Pyrenees, derived from instrumental records [25], comparable to the range found in our study. On the other hand, air temperatures were not significant in explaining diel temperature_range and temperature oscillation, which indicates an increase also in minimum water temperature during summer. Therefore, they would not be expected to change in the future.

Temperatures increase can drive changes in water bodies’ communities, such as functional traits, composition, biomass or abundance. For instance, in Canadian lakes, it was found that water temperatures were negatively related to zooplankton body size, a similar effect to the one produced by fish predation, and these combined effects would be non-additive [58]. Therefore, lakes warming would favour smaller zooplankton species. Combined fish predation with warming may increase small zooplankton by predation on large zooplankton [59], causing an increase in producers’ abundance due to less efficient consumption of small zooplankton [60]. Moreover, higher altitude lakes would be more sensitive to warming since they have less functional diversity than lower ones [58]. This functional diversity would move upwards, as species may change their distribution ranges to higher elevations [61]. At high elevations, cold stenothermal species are more vulnerable as they have a restricted distribution range [62], and they could be lost as a consequence of climate change [63]. A temperature increase can also advance zooplankton phenology [64]. Accumulated-degree-days can be modelled to predict recruitment and abundance of fish [65], which may be benefited by increasing accumulated degree-days. In this sense, in Pyrenean lakes, species, both natural and introduced, could develop in higher altitude lakes as a result of the accumulated-degree-days increase. The present paper opens the possibility to define the potential thermal habitat of lacustrine species in this mountain range. In contrast, maximum temperatures may induce thermal stress to certain species [10], influencing thus species composition [63]. These effects would have a more significant impact on higher water bodies where we have foreseen a greater increase in maximum temperatures. In addition, small increases of maximum temperatures in low and warm lakes may cause thermal stress in organisms, as their maximum temperatures are already high. Consequently, lower distribution of species in the Pyrenean range may be limited, while seeing their upper potential habitat increased. The knowledge about thermic variables and the models developed here enable making spatial extrapolations of water thermic variables, which are fundamental for disentangling ecological and conservation issues in a global change context.

## Supporting information

S1 Fig Morphological and radiation variables in the Pyrenees and studied lakes. Variables distribution in the Pyrenees (grey) and the studied lakes (black) for morphological and radiation variables in the water bodies. For the global of the Pyrenean water bodies, there is information of 3909 lakes and ponds, 2630 of which had data of their catchments, while the sample lakes include 59 lakes (See Table 1 for variables description. Here the variables are not transformed).
(DOCX)

S2 Fig Spring air temperatures in 14 Central and Eastern Pyrenean automatic weather stations from 2000 to 2015 given by Servei Meteorològic de Catalunya. Location of the automatic weather stations can be seen in Fig 1 and their information in S3 Table. A) temporal series B) correlogram of the series.
(DOCX)

S3 Fig Correlation between radiation variables. Global solar radiation (S), direct radiation (Dir), diffuse (Dif) and sun hours (Sun) are calculated for the lake (l), direct catchment (d) and total catchment (t).
(DOCX)

S4 Fig Pearson correlations between morphologic, radiation, and thermic variables of water bodies in the Pyrenees. See Table 1 for variables description.
(DOCX)

S1 Table Summary of the morphological, radiation and thermic variables in the Pyrenees and sampled water bodies. For the global of the Pyrenean water bodies, there is information of 3909 lakes and ponds, 2630 of which had data of their catchments, while the sample lakes include 59 lakes (See Table 1 for variables description. Here the variables are not transformed).
(DOCX)

S2 Table Data of the thermic and descriptive variables of the monitored Pyrenean lakes. Sample ID is a code to identify the temperature data in the lakes for each year. Lake ID is a code to identify each individual lake. The thermal variables of the lakes are: ADD7.6: Accumulated degree-days over 7.6°C (°C day); ADD4: Accumulated degree-days over 4.0°C (°C day); Tmean: Mean epilimnetic temperature of the ice free period (°C); Tmax: Maximum epilimnetic temperature (°C); DTR: Diel temperature range (°C); Tosc: Oscillation in maximum temperature in a three-day lapse (°C). Seasonal air temperatures are Tspring (°C) for spring (March, April and May)and Tsummer (°C) from summer (June, July and August). Descriptive variables are Year: The year of the temperature variables, Altitude (m); Larea (ha): Lake area; Dcatchment (ha): The catchment which flows directly to the lake; Tcatchment (ha): The total catchment of the lake; Radiation (h) The sun hours per day incoming to the lake catchment. X and Y (m) are the Geographic Coordinates in ETRS89 UTM 31N projection.
(XLSX)

S3 Table Description of the automatic weather stations. The geographic location (ETRS89 UTM 31N), the altitude and the date of functioning start of the automatic weather stations are detailed.
(DOCX)

S4 Table Summary table of the adjustment indicators of the models using different selection processes. In these cases the resulting models are multiple regression models without random structure, and therefore R^2^ ordinary and R^2^ adjusted are represented ^a^ (See Table 1 for variables description).
(DOCX)

S5 Table Table of the projections of the thermal variables of the Pyrenean lakes for the period between 2000 and 2019. The field Lake_ID includes a code for each individual lake. Register_ID includes a unique register for each lake and year. Also the year of the projections are detailed together with the thermal variables detailed in Table 1 and the geographic coordinates (ETRS89 UTM 31N).
(CSV)

S1 Section Accumulated degree-days calculation. (DOCX)
