# Supplementary material for: Factors of surface thermal variation in high-mountain lakes of the Pyrenees
Source: PLoS One. 2021 Aug 3;16(8):e0254702. doi: 10.1371/journal.pone.0254702 (PMC8330907; doi:10.1371/journal.pone.0254702)
Supplement: S1 Fig — Variables distribution in the Pyrenees (grey) and the studied lakes (black) for morphological and radiation variables in the water bodies. For the global of the Pyrenean water bodies, there is information of 3909 lakes and ponds, 2630 of which had data of their catchments, while the sample lakes include 59 lakes (See Table 1 for variables description. Here the variables are not transformed). (DOCX) [file pone.0254702.s001.docx]

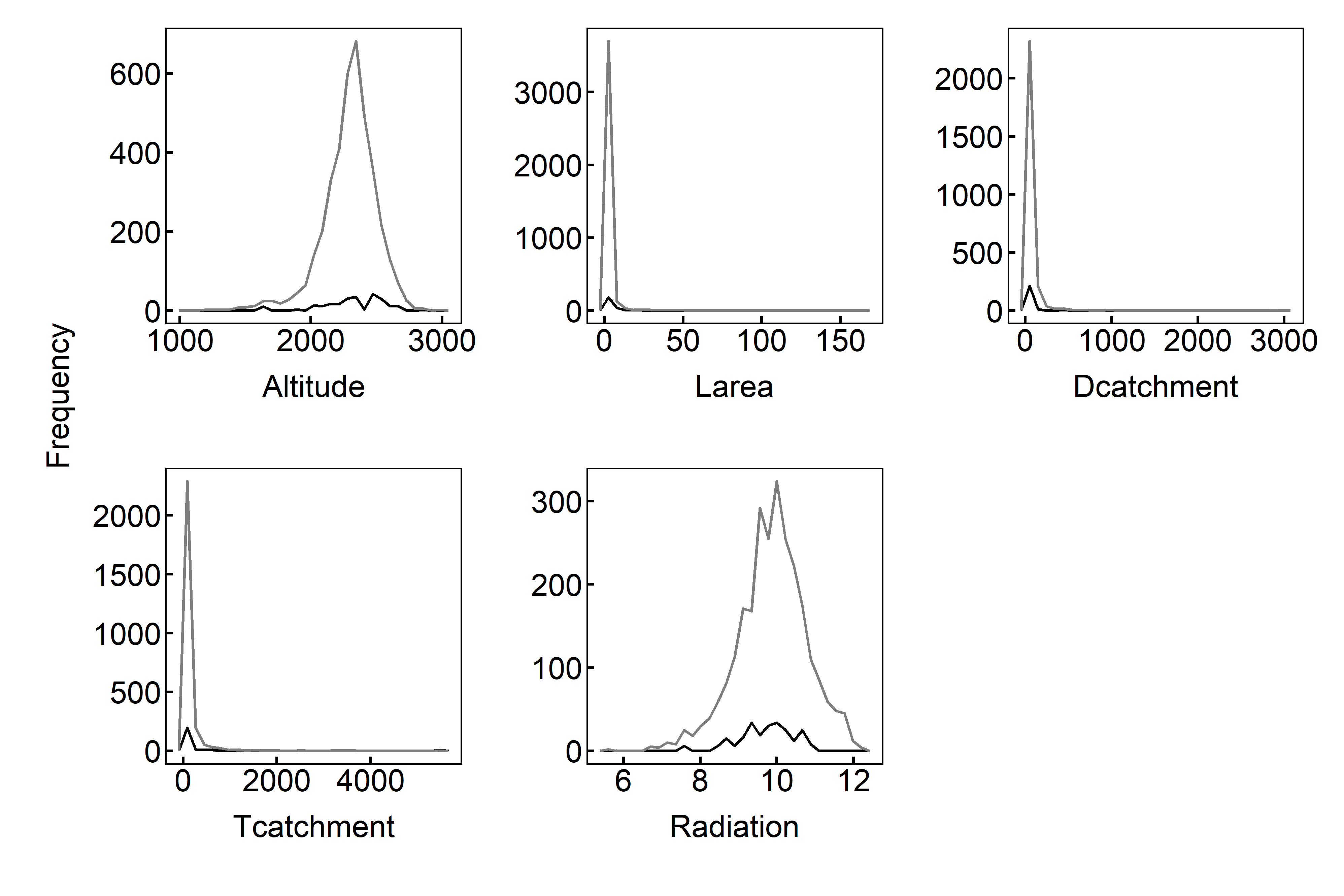
**S1 Fig.** **Morphological and radiation variables in the Pyrenees and studied lakes.**

Variables distribution in the Pyrenees (grey) and the studied lakes (black) for morphological and radiation variables in the water bodies. For the global of the Pyrenean water bodies, there is information from 3909 lakes and ponds, for which 2630 had data for their catchments, while the sample lakes include 59 lakes (See Table 1 for variables description, here the variables are not transformed).
