# Supplementary material for: Factors of surface thermal variation in high-mountain lakes of the Pyrenees
Source: PLoS One. 2021 Aug 3;16(8):e0254702. doi: 10.1371/journal.pone.0254702 (PMC8330907; doi:10.1371/journal.pone.0254702)
Supplement: S2 Fig — Location of the automatic weather stations can be seen in Fig 1 and their information in S3 Table. A) temporal series B) correlogram of the series. (DOCX) [file pone.0254702.s002.docx]

**S2 Fig.** **Spring air temperatures in 14 Central and Eastern Pyrenean AWS from 2000 to 2015 given by Servei Meteorològic de Catalunya.**


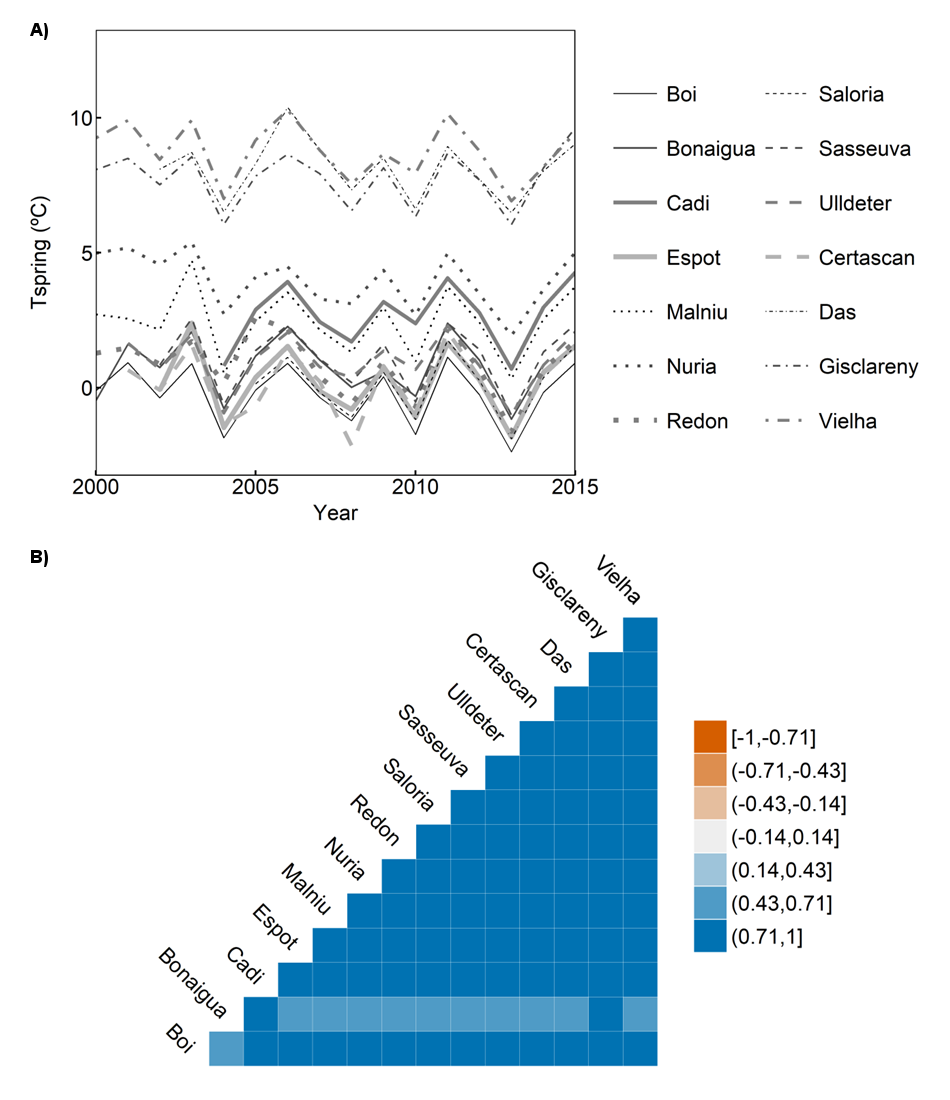


Location of the AWS can be seen in Fig. 1 and their information in S4 Table. A) temporal series B) correlogram of the series.
