# Supplementary material for: Factors of surface thermal variation in high-mountain lakes of the Pyrenees
Source: PLoS One. 2021 Aug 3;16(8):e0254702. doi: 10.1371/journal.pone.0254702 (PMC8330907; doi:10.1371/journal.pone.0254702)
Supplement: S3 Fig — Global solar radiation (S), direct radiation (Dir), diffuse (Dif) and sun hours (Sun) are calculated for the lake (l), direct catchment (d) and total catchment (t). (DOCX) [file pone.0254702.s003.docx]

**
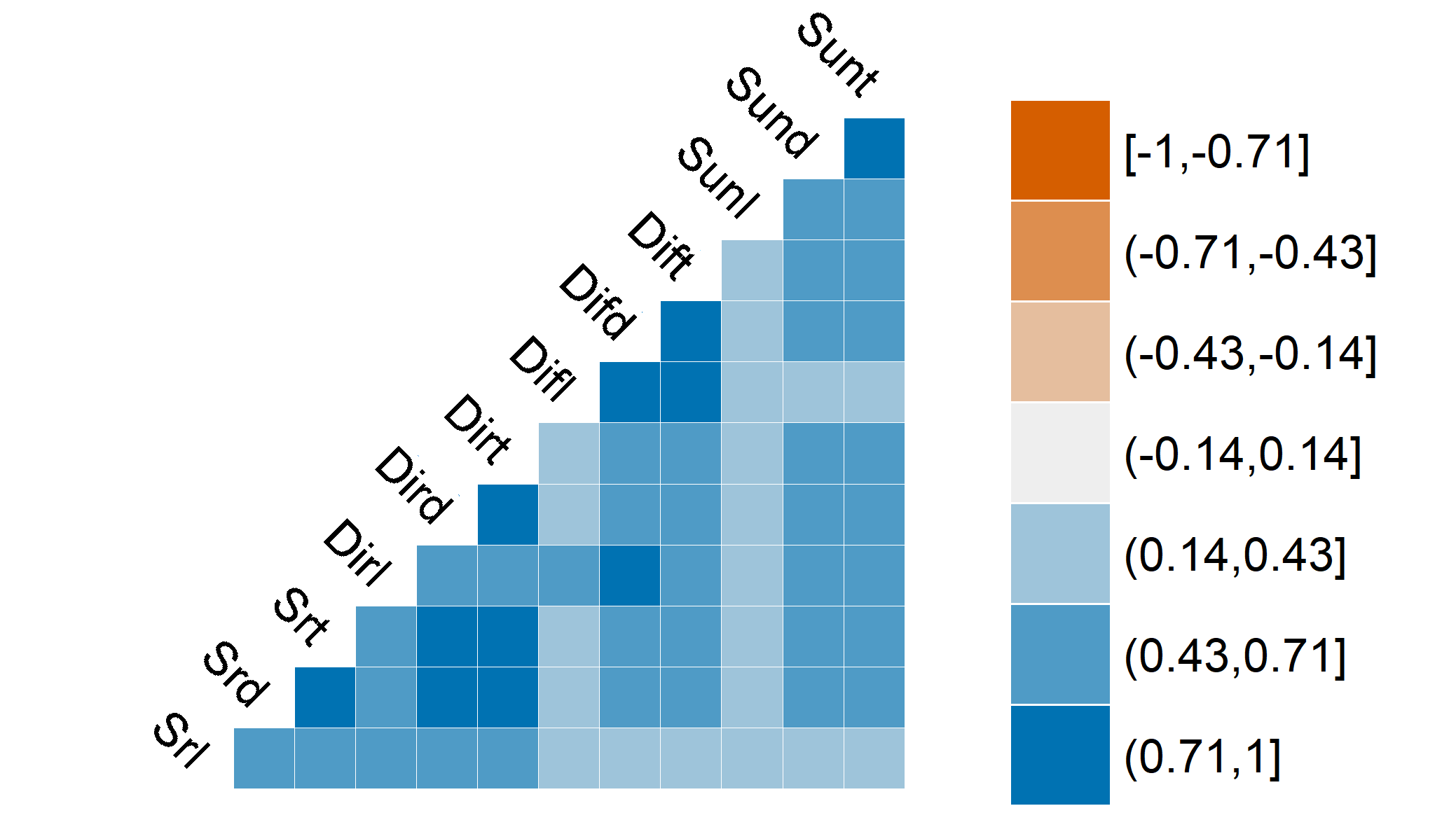
S3 Fig.** **Correlation between radiation variables.**

Global solar radiation (S), direct radiation (Dir), diffuse (Dif) and sun hours (Sun) are calculated for the lake (l), direct catchment (d) and total catchment (t).
