# Supplementary material for: Factors of surface thermal variation in high-mountain lakes of the Pyrenees
Source: PLoS One. 2021 Aug 3;16(8):e0254702. doi: 10.1371/journal.pone.0254702 (PMC8330907; doi:10.1371/journal.pone.0254702)
Supplement: S4 Fig — See Table 1 for variables description. (DOCX) [file pone.0254702.s004.docx]

**
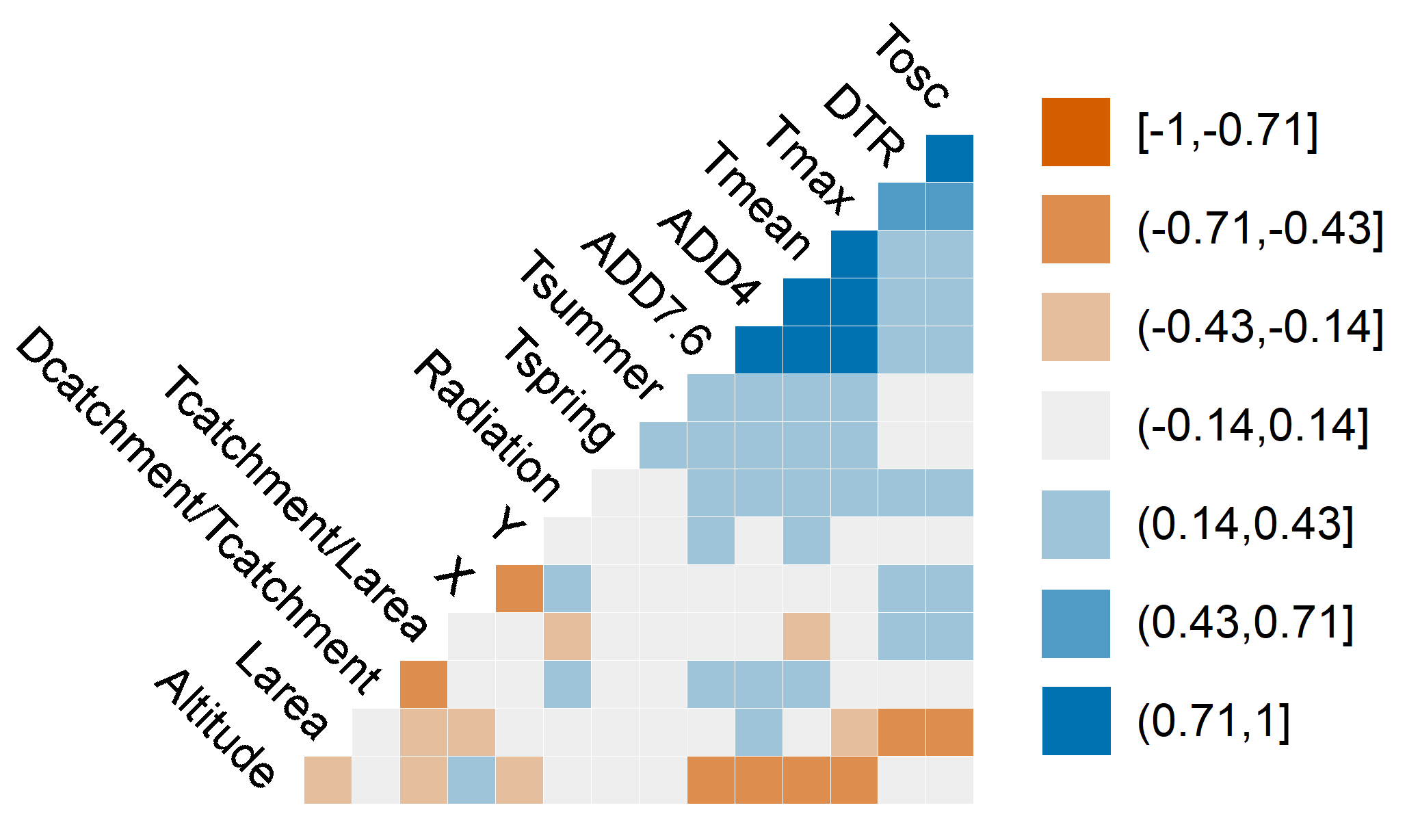
S4 Fig.** **Pearson correlations between morphologic, radiation, and thermic variables of water bodies in the Pyrenees.**

See Table 1 for variables description.
