# Supplementary material for: Factors of surface thermal variation in high-mountain lakes of the Pyrenees
Source: PLoS One. 2021 Aug 3;16(8):e0254702. doi: 10.1371/journal.pone.0254702 (PMC8330907; doi:10.1371/journal.pone.0254702)
Supplement: S3 Table — The geographic location (ETRS89 UTM 31N), the altitude and the date of functioning start of the automatic weather stations are detailed. (DOCX) [file pone.0254702.s007.docx]

**S3 Table.** **Description of the automatic weather stations (AWS).**

The geographic location (ETRS89 UTM 31N), the altitude and the date of functioning start of the AWS are detailed.

| Weather station | Latitude (º) | Longitude (º) | Altitude (m) | Function date |
| --- | --- | --- | --- | --- |
| Vielha | 42.7 | 0.79 | 1002 | 15/02/1996 |
| Das | 42.39 | 1.87 | 1097 | 22/05/2001 |
| Gisclareny | 42.26 | 1.76 | 1386 | 12/03/1999 |
| Núria | 42.4 | 2.16 | 1971 | 15/05/1998 |
| Cadí | 42.29 | 1.71 | 2143 | 06/11/2003 |
| Sasseuva | 42.77 | 0.73 | 2228 | 20/09/2001 |
| Malniu | 42.47 | 1.78 | 2230 | 03/11/1999 |
| Lac Redon | 42.64 | 0.78 | 2247 | 15/07/1999 |
| Bonaigua | 42.65 | 0.98 | 2266 | 06/11/1997 |
| Certascan | 42.7 | 1.27 | 2400 | 11/11/2000 |
| Ulldeter | 42.42 | 2.25 | 2410 | 28/09/2011 |
| Salòria | 42.52 | 1.37 | 2451 | 30/09/2004 |
| Espot | 42.53 | 1.05 | 2519 | 01/03/2002 |
| Boí | 42.47 | 0.88 | 2535 | 13/11/1998 |
