# Supplementary material for: Factors of surface thermal variation in high-mountain lakes of the Pyrenees
Source: PLoS One. 2021 Aug 3;16(8):e0254702. doi: 10.1371/journal.pone.0254702 (PMC8330907; doi:10.1371/journal.pone.0254702)
Supplement: S4 Table — In these cases the resulting models are multiple regression models without random structure, and therefore R2 ordinary and R2 adjusted are represented a (See Table 1 for variables description). (DOCX) [file pone.0254702.s008.docx]

**S4 Table. Summary table of the fitting indicators of the models using different selection processes.**

In these cases the resulting models are multiple regression models without random structure, and therefore R^2^ ordinary and R^2^ adjusted are represented ^a^ (See Table 1 for variables description).

| Model | | AIC | BIC | RMSE | R^2^ marginal/ ^a^ordinary | R^2^ conditional/^a^adjusted | Random structure | Fixed structure |
| --- | --- | --- | --- | --- | --- | --- | --- | --- |
| ADD7.6 | forward interaction | 318 | 352 | 179.3 | 0.74 | 0.83 | water body/year | Radiation+Tcatchment/Larea+Larea+Altitude+Tspring+ Larea:Altitude |
| ADD7.6 | forward no interaction | 331 | 362 | 190.9 | 0.71 | 0.82 | water body/year | Radiation+Tcatchment/Larea+Larea+Altitude+Tspring |
| ADD7.6 | backward no interaction | 331 | 362 | 190.9 | 0.71 | 0.82 | water body/year | Radiation+Tcatchment/Larea+Larea+Altitude+Tspring |
| ADD7.6 | backward interaction | 322 | 363 | 175.2 | 0.75 | 0.84 | water body/year | Radiation+Tcatchment/Larea+X+Larea+Altitude+Tspring+Larea:Altitude+Altitude:Tspring |
| ADD4 | forward interaction | 300 | 336 | 232.6 | 0.75 | 0.82 | water body/year | Radiation+Tcatchment/Larea+Larea+Altitude +Tspring+Larea:Altitude |
| ADD4 | forward no interaction | 300 | 330 | 248.2 | 0.71 | 0.82 | water body/year | Radiation+Tcatchment/Larea+Larea +Altitude+Tspring |
| ADD4 | backward no interaction | 302 | 335 | 244.9 | 0.72 | 0.82 | water body/year | Radiation+Tcatchment/Larea+X+Larea+Altitude+Tspring |
| ADD4 | backward interaction | 301 | 334 | 234.9 | 0.72 | 0.83 | year | Radiation+Tcatchment/Larea+Y+Larea+Altitude+Tspring+Larea:Altitude |
| Tmean | forward interaction | 493 | 530 | 1.1 | 0.52 | 0.63 | water body | Radiation+Dcatchment/Tcatchment +Tcatchment/Larea+Tsummer+Tspring+Larea+Altitude+ Larea:Altitude |
| Tmean | forward no interaction | 504 | 545 | 1.3 | 0.46 | 0.66 | water body | Radiation+Dcatchment/Tcatchment+Tcatchment/Larea+X+Y+Larea+Altitude+Tspring+Tsummer |
| Tmean | backward no interaction | 504 | 545 | 1.3 | 0.46 | 0.66 | water body | Radiation+Dcatchment/Tcatchment+ Tcatchment/Larea+X+Y+Larea+Altitude+Tspring+ Tsummer |
| Tmean | backward interaction | 493 | 530 | 1.1 | 0.52 | 0.63 | water body | Radiation+Dcatchment/Tcatchment+Tcatchment/Larea+ Tsummer+Tspring+Larea+Altitude+Larea:Altitude |
| Tmax | forward interaction | 395 | 425 | 1.9 | 0.58 | 0.77 | year | Radiation+Tcatchment/Larea+Larea+Altitude+ Larea:Altitude+Altitude:Tspring |
| Tmax | forward no interaction | 407 | 431 | 2.0 | 0.55 | 0.74 | year | Radiation+Tcatchment/Larea+Larea+Altitude |
| Tmax | backward no interaction | 409 | 436 | 2.0 | 0.55 | 0.75 | year | Radiation+Dcatchment/Tcatchment+Tcatchment/Larea+ Larea+Altitude |
| Tmax | backward interaction | 399 | 437 | 1.9 | 0.60 | 0.78 | year | Radiation+dc.tc+Tcatchment/Larea+X+Larea+Altitude+ Larea:Altitude+Altitude:Tspring |
| DTR | forward interaction | 542 | 563 | 0.55 | 0.45^a^ | 0.44^a^ | - | Radiation + X + Larea + Altitude + Larea:Altitude |
| DTR | forward no interaction | 544 | 568 | 0.56 | 0.43^a^ | 0.42^a^ | - | Radiation + X + Larea + Altitude |
| DTR | backward no interaction | 523 | 544 | 0.56 | 0.43^a^ | 0.42^a^ | - | Radiation + X + Larea + Altitude |
| DTR | backward interaction | 523 | 544 | 0.55 | 0.45^a^ | 0.44^a^ | - | Radiation + X + Larea + Altitude + Larea:Altitude |
| Tosc | forward interaction | 508 | 528 | 0.26 | 0.50 | 0.53 | year | Radiation+Larea+Altitude |
| Tosc | forward no interaction | 508 | 528 | 0.26 | 0.50 | 0.53 | year | Radiation+Larea+Altitude |
| Tosc | backward no interaction | 508 | 528 | 0.26 | 0.50 | 0.53 | year | Radiation+Larea+Altitude |
| Tosc | backward no interaction | 512 | 542 | 0.26 | 0.47 | 0.54 | water body/year | Radiation+X+Larea+alt+Larea:alt |
