# Supplementary material for: Factors of surface thermal variation in high-mountain lakes of the Pyrenees
Source: PLoS One. 2021 Aug 3;16(8):e0254702. doi: 10.1371/journal.pone.0254702 (PMC8330907; doi:10.1371/journal.pone.0254702)
Supplement: S1 Section — (DOCX) [file pone.0254702.s010.docx]

**S1 Section. Accumulated degree-days calculation.**

First, the daily heat accumulation (degree-days; DD) were calculated from daily maximum and minimum temperatures and assuming the single sine curve as an approximation of the diurnal temperature curve (1). Degree-days were only calculated when maximum temperature was above T_T_. Two different formulae were used for the calculation (2) When the minimum temperature (Tmin) was greater than T_T_, the DD were calculated from the difference between mean daily temperature and T_T_:

$T_{min} > T_{T} \to DD= \frac{T_{max}+T_{min}}{2} - T_{T}$ (1)

When minimum temperature was below T_L_ the formula used was:

${T_{min} <T}_{T}<T_{max}\to DD=\frac{1}{\pi}\left[ \left( \frac{T_{max}+T_{min}}{2}-T_{T} \right)\left( \frac{\pi}{2}-\theta_{1} \right) \right]+Acos(\theta_{1})$ (2)

Where:

$$\theta_{1}=arcsin\left[ \left( T_{T}- \frac{T_{max}+T_{min}}{2} \right)\div A \right]$$

$$A= \frac{T_{max}-T_{min}}{2}$$

Once the daily DD were calculated for each water body, we calculated the ADD during the ice-free period by adding DD values. Finally, we transformed the ADD measured at 1.5 m to those at 0.1 m, which is a more representative depth for many aquatic organisms habitat. The transformation was done using linear relationships of ADD at both depths, which were adjusted with data from five lakes for which we had measures; (ADD7.6_0.1 = 1.10 ADD7.6_1.5 + 15.83; R^2^ = 0.99, p < 0.001) and (ADD4_0.1 = 1.02 ADD4_1.5 + 41.56; R^2^ = 0.99, p < 0.001).

**References**

1. Zalom FG, Goodell P, Wilson L, Barnett WW, Bentley WJ. Degree-Days, the Calculation and Use of Heat Units in Pest Management: . University of California Division of Agriculture and Natural Resources Leaflet 21373; 1984.

2. Baskerville GL, Emin P. Rapid estimation of heat accumulation from maximum and minimum temperatures. Ecology. 1969;50(3):514–7.
